# Accurate Bounds on Lyapunov Exponents for Expanding Maps of the Interval

**DOI:** 10.1007/s00220-022-04495-7

**Published:** 2022-11-17

**Authors:** M. Pollicott, P. Vytnova

**Affiliations:** 1 Department of Mathematics, Warwick University, Coventry, CV4 7AL UK; 2 Department of Mathematics, University of Surrey, Guildford, GU2 7XH UK

## Abstract

In this short note we describe a simple but remarkably effective method for rigorously estimating Lyapunov exponents for expanding maps of the interval. We illustrate the applicability of this method with some standard examples.

## Introduction

Lyapunov exponents give a well known characterization of the instability in a dynamical system by quantifying how nearby orbits separate. In particular, a non-zero Lyapunov exponent with respect to an invariant ergodic measure implies that typical nearby orbits separate exponentially quickly. It is therefore useful to have a rigorous and effective estimate of these values, in particular, in the setting of one dimensional expanding maps for an absolutely continuous invariant probability measure. This problem has attracted the attention of many authors who have employed a variety of different methods (see [5, 10, 13, 22]).

In this paper we will consider the nice class *NC* of expanding piecewise analytic mixing Markov maps of the interval. We recall the definition.

### Definition 1.1

Let $I = [a,b]$ be a closed interval. We say that a map *f* belongs to the class *NC*(*I*) if there exists a partition $a=x_1< x_2< \cdots < x_{n+1} = b$ such that:

1. The restrictions $f|_{[x_j, x_{j+1}]}$ are analytic maps for $j = 1, \ldots n$;
2. There exists $d>1$ such that for all $1\le k \le n$ and for all $x \in (x_k, x_{k+1})$ we have $|f'(x)| \ge d$.
3. The Markov property holds: if $f((x_k,x_{k+1})) \cap (x_j,x_{j+1}) \ne \varnothing$; then $f( (x_k,x_{k+1})) \supset (x_j,x_{j+1})$.
4. The map *f* is topologically mixing^1^ (i.e., for any non-empty open sets $U,V \subset I$ there exists $n_0\ge 1$ such the for all $n \ge n_0$ we have $U\cap f^{-n}V \ne \varnothing$).

For every map $f \in NC(I)$ there exists a unique absolutely continuous *f*-invariant probability measure $d\mu = \rho (x) dx$ on *I* [7]. In particular, the measure $\mu$ is ergodic. Furthermore, every map of the class *NC*(*I*) is invertible on each of the intervals $(x_j,x_{j+1})$, in particular, there exist analytic maps $f_{jk}:(x_j, x_{j+1}) \rightarrow (x_k, x_{k+1})$, such that $f (f_{jk}(x)) = x$ whenever $f((x_k,x_{k+1})) \cap (x_j,x_{j+1}) \ne \varnothing$. The maps $f_{jk}$ are called *inverse branches* of *f*.

A standard approach to constructing the measure $\mu$ is to use transfer operators. Let us denote by ${\mathcal {B}}$ the space of analytic functions on the disjoint union $\coprod _{k=1}^n[x_k,x_{k+1}]$. We can introduce a one-parameter family of linear operators ${\mathcal {L}}_t: {\mathcal {B}} \rightarrow {\mathcal {B}}$ ($t \in {\mathbb {R}}$) called *transfer operators* defined in terms of inverse branches of *f*:

1 $$\begin{aligned}{}[{\mathcal {L}}_t h](x) = \sum _{k: f((x_k,x_{k+1})) \cap (x_j,x_{j+1}) \ne \varnothing } |f_{jk}^\prime (x)|^t h(f_{jk}(x))\chi _{[x_j, x_{j+1}]}(x), \end{aligned}$$

where $\chi _{[x_j, x_{j+1}]}$ is the indicator function of the interval $[x_j, x_{j+1}]$. In the special case that *f* is full branched, i.e., $f((x_k,x_{k+1})) = I$ for $k=1, \ldots , n$, we can denote the inverses $f_k: I \rightarrow (x_k, x_{k+1})$, i.e., $f(f_k(x))=x$ for all $a \le x \le b$. The transfer operators ${\mathcal {L}}_t: C^\omega (I) \rightarrow C^\omega (I)$ ($t \in {\mathbb {R}}$) then take the form

2 $$\begin{aligned}{}[{\mathcal {L}}_t h](x) = \sum _{j=1}^n |f_j^\prime (x)|^t h(f_j(x)), \qquad x \in I. \end{aligned}$$

Most of our examples will be of this type.

It is well known [7] that the positive density $\rho \in C^\omega (I)$ of the measure $\mu$ is characterized as a fixed point for the operator ${\mathcal {L}}_1$, corresponding to the parameter choice $t=1$. Nevertheless, including this operator into a one parameter family will serve us well later.

We can now define the Lyapunov exponent of the system $(I,f,\mu )$ which quantifies the sensitivity of typical orbits on initial conditions.

### Definition 1.2

We define the *Lyapunov exponent* for the map *f* and its stationary measure $\mu$ by

$$\begin{aligned} \lambda (f, \mu ) = \int _I \log |f'(x)| d\mu (x). \end{aligned}$$

### Remark 1.3

This value coincides with the metric entropy $h(\mu )$ of the measure $\mu$ by the Rokhlin’s formula [17].

Since the measure $\mu$ is ergodic, applying the Birkhoff ergodic theorem one can see that for $\mu$-almost all $x\in I$ we get

$$\begin{aligned} \lim _{n \rightarrow +\infty } \frac{1}{n} \log |(f^n)'(x)| = \lambda (f, \mu ). \end{aligned}$$

There are various methods used to estimate the Lyapunov exponents. Probably the most famous are Ulam’s method and finite section methods [13]. Another approach is based on periodic points method [11]. Recent work by Wormell [22] is based on the Galerkin spectral method originally developed for PDEs. In this note we present an alternative approach, which starts with the *spectral Chebyshev collocation method*, also initially developed for PDEs [6, §3]. In dynamical systems it has been used succsefully by Babenko and Yuriev in their solution of the Gauss problem [1] and by Babenko in his computation of the fixed point of the renormalisation operator for the period-doubling map [2]. More recently, Bandtlow and Slipantschuk [4] applied the spectral Chebyshev collocation method in the setting of holomorphic contractions and showed it to be an effective approach to obtaining the spectral data of the transfer operator. In constrast to our approach in [4] the error is estimated directly using the spectral properties of the transfer operator.

In our approach, we combine the Chebyshev collocation method with a small amount of thermodynamic formalism (involving the pressure function) and a classical min-max method. The main advantage of this combination of ideas is that it provides an efficient and effective way to estimate Lyapunov exponents and gives rigorous estimates with validated error bounds.

The main results we present in this note are the following. The first theorem gives a method for obtaining rigorous bounds on the Lyapunov exponent.

### Theorem 1.4

Let $f:I\rightarrow I$ be an expanding piecewise analytic mixing Markov map of the interval with absolutely continuous probability measure $\mu$. Assume that for some $\varepsilon > 0$ there exists a pair of positive functions^2^ $p, q :I \rightarrow {\mathbb {R}}^{+}$ and a pair of numbers $0< \alpha < \beta$ such that

3 $$\begin{aligned} \sup _I\frac{ {\mathcal {L}}_{1+ \varepsilon } p}{ p } \le e^{-\alpha } \hbox { and } \sup _I\frac{ {\mathcal {L}}_{1- \varepsilon } q}{ q } \le e^{\beta }. \end{aligned}$$

Then the following double inequality holds:

$$\begin{aligned} \frac{\alpha }{\varepsilon } \le \lambda (f, \mu ) \le \frac{\beta }{\varepsilon }. \end{aligned}$$

### Remark 1.5

The idea behind Theorem 1.4 is that for the class of maps we consider for any positive function *p* the supremum of the ratio $\frac{{\mathcal {L}}_tp}{p}$ gives an upper bound on the leading eigenvalue of ${\mathcal {L}}_t$.

Note that if the function *p* is close to the leading eigenfunction of the operator ${\mathcal {L}}_{t}$ then the ratio $\frac{\mathcal L_{t} p}{p}$ is close to a constant function. This observation allows us to estimate the ratios rigorously in practice.

### Remark 1.6

If we do not assume that *f* is Markov then the statement of the Theorem remains true, however, in this setting the construction of the functions *p* and *q* is more challenging since the eigenfunctions of ${\mathcal {L}}_t$ might be non-analytic (but of bounded variation). As we will see later, in practical applications, the interval $(\alpha ,\beta ) \ni \lambda (f,\mu )$ depends on the quality of approximation of the leading eigenfunction of $\mathcal L_t$ by polynomials *p* and *q*.

The next theorem guarantees that the previous theorem can be used to get bounds on the Lyapunov exponent which are arbitrary accurate. Note that Theorem 1.4 also holds under the weaker assumption that $f:I\rightarrow I$ is an expanding piecewise $C^2$ mixing Markov map of the interval, however, in this case it is much harder to compute the functions *p* and *q* which will give us good estimates on the Lyapunov exponent. In addition, it is convenient to assume analyticity in order to apply the following theorem.

### Theorem 1.7

Let $f:I\rightarrow I$ be an expanding piecewise analytic mixing Markov map of the interval with absolutely continuous probability measure $\mu$. Then for any $\delta > 0$ we can choose $\varepsilon > 0$, $0< \alpha < \beta$ and strictly positive polynomials $p, q : I \rightarrow {\mathbb {R}}$ satisfying (3) with

4 $$\begin{aligned} \left| \frac{\beta }{\varepsilon } - \frac{\alpha }{\varepsilon }\right| < \delta . \end{aligned}$$

## Examples

In this section we will demonstrate how Theorem 1.4 can be used in practice. To this end we consider four examples, and compare the estimates we obtain for the Lyapunov exponents with previously known results.

Theorem 1.4 allows us to obtain rigorous bounds using the built-in MaxValue routine in Mathematica, and the implementation is relatively straightforward. However, some care is required in choosing parameters during the construction of the functions *p* and *q*. In Sect. 4.2 we give more details on the practicalities of the implementation.

### Classical example: the Lanford map

**Fig. 1:**
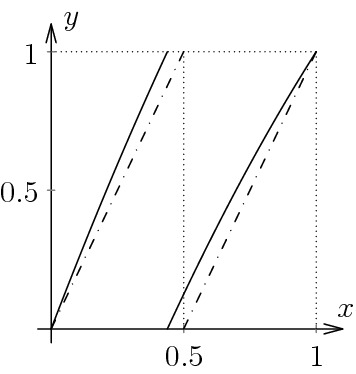
The solid curve is a plot of the Lanford map. For comparison, the dashed line is the plot of the doubling map

We will first illustrate our approach with the standard example of the Lanford map [14] (see also [3]). The original Lanford map $f_L:[0,1] \rightarrow [0,1]$ is defined by

5 $$\begin{aligned} f_L(x) = 2x + \frac{1}{2}x(1-x) \quad \mod 1 \end{aligned}$$

and the graph of $f_L$ is shown in Fig. 1. Observe that the map is uniformly expanding with $T'(x) \ge T'(1) = \frac{3}{2}$ for $0 \le x \le 1$.

The inverse branches of $f_L$ are contractions given by

$$\begin{aligned} f_1(x) = \frac{5 - \sqrt{25 - 8x}}{2} \quad \text{ and } \quad f_2(x) = \frac{5 - \sqrt{17 - 8x}}{2}. \end{aligned}$$

The transfer operator ${\mathcal {L}}_t$ therefore takes the form

$$\begin{aligned} {[}{\mathcal {L}}_th](x) = \left( \frac{2}{ \sqrt{25 - 8x}}\right) ^t h\left( \frac{5 - \sqrt{25 - 8x}}{2} \right) + \left( \frac{2}{\sqrt{17 - 8x}}\right) ^t h\left( \frac{5 - \sqrt{17 - 8x}}{2} \right) . \end{aligned}$$

Due to simplicity of the formulae involved we shall attempt to obtain estimates on the Lyapunov exponents of particularly high accuracy, to demonstrate the power of our method. Namely, we shall choose $\varepsilon = 10^{-180}$. Then we fix $N=400$ and compute the nodes of the Chebyshev polynomial $T_{400}$ to 512-digits precision. Subsequently, we want to construct the functions *p* and *q* as polynomials of degree 399 using the spectral Chebyshev collocation method. We validate that the polynomials *p* and *q* are positive using the method described in Sect. 4.2. At this point we apply the built-in MaxValue Mathematica routine to calculate

$$\begin{aligned} \alpha : = -\log \left( {\texttt {MaxValue}} \frac{\mathcal L_{1+\varepsilon } p}{p}\right) \text{ and } \beta : = \log \left( {\texttt {MaxValue}} \frac{{\mathcal {L}}_{1-\varepsilon } q}{q} \right) . \end{aligned}$$

We obtain the following values:

$$\begin{aligned} \alpha = 6.&5766178000\,6597677541\,5824138238\,3206574324\,1069580012\,2019539528\, \\&0269163266\,6111554023\,7595564597\,5291517482\,9642156331\,7980263014\, \\&8859489891 \times 10^{-181} \text{ and } \end{aligned}$$

$$\begin{aligned} \beta = 6.&5766178000\,6597677541\,5824138238\,3206574324\,1069580012\,2019539528\, \\&0269163266\,6111554023\,7595564597\,5291517482\,9642156331\,7980263014\, \\&8859489094 \times 10^{-181}; \end{aligned}$$

which are each presented to 130 significant figures. In particular, with these choices Theorem 1.4 yeilds that $\varepsilon ^{-1} \alpha< \lambda (f_L,\mu ) < \varepsilon ^{-1}\beta$ therefore

$$\begin{aligned} \begin{aligned} \lambda (f_L, \mu ) =\,&0.65766178000\,6597677541\,5824138238\,3206574324\,1069580012\\ {}&2019539528\,0269163266\,6111554023\,7595564597\,5291517482\,\\ {}&9642156331\,7980263014\,88594891 \pm 10^{-128}\\\end{aligned} \end{aligned}$$

This value has previously been computed by Wormell [22] and her result agrees with the above. See also [10, §10.1]. In the present approach, the simplicity of the functions *p* and *q* is the source of the efficiency of the approach. In particular, this estimate was obtained in approximately 2 hours on a *personal* Macbook pro laptop with 2.8 GHz Quad-Core Intel Core i7 and 16 GB 2133 MHz LPDDR3 using Mathematica.

#### Remark 2.1

In addition to using the internal MaxValue function, whose code is not available to the public, we can apply a simple Monte-Carlo type method to numerically verify the value we obtained. More precisely, we generate $N_{mc}=1000$ pseudo-random points $x_j$, $j=1, \ldots , 1000$ in the interval [0, 1] and evaluate both ratios at these points to get the values

$$\begin{aligned} y_j^{+}: = \frac{[{\mathcal {L}}_{1+\varepsilon } p](x_j)}{p(x_j)} \qquad \text{ and } \qquad y_j^{-}: = \frac{[{\mathcal {L}}_{1-\varepsilon } q](x_j)}{q(x_j)}, \quad j = 1,\ldots ,100. \end{aligned}$$

Then we compute $a_1:=-\log \max _j y_j^{+}$ and $b_1:=\log \max _j y_j^{-}$. Repeating this procedure a total of $t_{mc}=100$ times, we obtain the values that are within a distance of $10^{-345}$ from $\alpha$ and $\beta$, respectively. In particular, we see that our estimate agrees with the estimate given by the function MaxValue.

### Lanford family of maps

We can extend the first example by including it in a larger family of maps. More precisely, we can include the Lanford map (5) into a family of expanding maps $f_c:[0,1] \mapsto [0,1]$ defined by

$$\begin{aligned} f_c(x) := 2x + c x(1-x) \quad \mod 1, \qquad 0< c <1. \end{aligned}$$

Observe that for the chosen parameter values $f_c'(x) = 2 + c (1-2x) \ge 2 - c = f_c'(1)$ for $0 \le x \le 1$ and so the map $f_c$ is expanding. Then the inverse branches $f_1, f_2 :I \rightarrow I$ are contractions defined by

$$\begin{aligned} f_1(x) = \frac{2+ c - \sqrt{(2+c)^2 - 4 c x}}{2 c } \quad \text{ and } \quad f_2(x) = \frac{2+ c - \sqrt{(2+ c)^2 - 4 c (x+1)}}{2c }. \end{aligned}$$

Following the formula (1) we obtain the associated transfer operator ${\mathcal {L}}_t$:

$$\begin{aligned} \begin{aligned} \left[ {\mathcal {L}}_t h\right] (x) =&\left( \frac{1}{\sqrt{(2+ c )^2 - 4 c x}}\right) ^t \cdot h\left( \frac{2 + c - \sqrt{(2+ c )^2- 4 c x}}{2 c } \right) \\&+ \left( \frac{1}{\sqrt{(2+ c )^2 - 4 c (x+1)}}\right) ^t \cdot h\left( \frac{2 + c - \sqrt{(2+ c )^2- 4 c (x+1)}}{2 c } \right) . \end{aligned} \end{aligned}$$

We next want to compute the Lyapunov exponent $\lambda (c):=\lambda (f_c,\mu _c)$ for forty equally spaced values $c = c_j = 0.001+\frac{j-1}{40}$, with $j = 1,\dots ,40$ with an error of $10^{-3}$ to sketch a graph of $\lambda$ as a function of *c*. For this purpose we choose $\varepsilon =10^{-3}$ and $m=60$ and compute the nodes of the Chebyshev polynomial $T_{60}$ with accuracy of 256 digits. We then apply Theorem 1.4 and obtain lower and upper bounds for the Lyapunov exponent. The precision of the MaxValue routine in the computation was set to 128 digits.

**Fig. 2:**
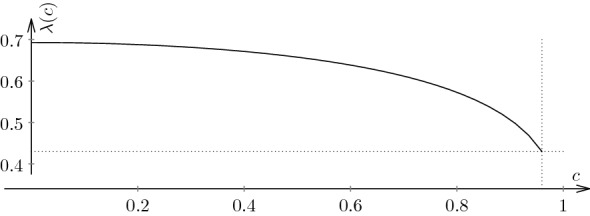
A plot of the Lyapunov exponent for the Lanford family based on the calculation for the 40 parameter values in the interval [0.001, 0.99]. The dependence of the Lyapunov exponent on *c* is analytic. It appears that the derivative $\frac{d \lambda (f_c,\mu _c)}{d c} \rightarrow -\infty$ as $c \rightarrow 1$. At the other end we have $\lambda (f_c,\mu _c) \rightarrow \log 2 = 0.693\ldots$ as $c \rightarrow 0$ which is expected, since $c=0$ corresponds to the doubling map

Based on this calculation, we sketch the functions $$\frac{\alpha (c)}{\varepsilon }$$ (dashed curve) and $$\frac{\beta (c)}{\varepsilon }$$ (solid curve) in Fig. 2. We see that for $$0.01< c < 0.96$$ the two curves are indistinguishable. However in the interval $$ 0.96< c <0.99$$ they appear to be different. This reflects the fact that $$f_c^\prime (1)\rightarrow 1$$ as $$c\rightarrow 1$$, i.e. the map $$f_c$$ has weak hyperbolicity for *c* close to 1. Uniform hyperbolicity is essential for Theorems 1.4 and 1.7 to be applicable.

In addition, we may also calculate the Lyapunov exponent for a selected parameter value $c = \frac{1}{4}$, for example, with high accuracy. To this end, we choose $\varepsilon =10^{-180}$ and compute 300 zeros of the Chebyshev polynomial $T_{300}$ with accuracy of 400 digits. Then we apply the spectral collocation method to construct polynomials *p* and *q* of degree 299. As before, we verify this this functions are positive, and apply MaxValue with working precision 400.

$$\begin{aligned} \alpha : = -\log \left( {\texttt { MaxValue}} \frac{\mathcal L_{1+\varepsilon } p}{p}\right) \text{ and } \beta : = \log \left( {\texttt { MaxValue}} \frac{{\mathcal {L}}_{1-\varepsilon } q}{q} \right) . \end{aligned}$$

We obtain the following values (for which we give 166 digits):

$$\begin{aligned} \alpha =0.&6851020685\,7610906837\,8941120635\,3368474791\,2954208389\,7263352003\,\\&7686275679\,0996831645\,2222918013\,3822749913\,1527755618\,1523970004\,\\&1829353798\,5819153203\,8804954205\,2390123411\,591687 \times 10^{-180}; \text{ and } \\ \beta =0.&6851020685\,7610906837\,8941120635\,3368474791\,2954208389\,7263352003\,\\&7686275679\,0996831645\,2222918013\,3822749913\,1527755618\,1523970004\,\\&1829353798\,5819153203\,8804954205\,2390123411\,591699 \times 10^{-180}. \end{aligned}$$

This gives the value of the Lyapunov exponent with accuracy of 164 decimal places:

$$\begin{aligned} \lambda \left( f_{\frac{1}{4}},\mu _{\frac{1}{4}}\right) =\,&0.6851020685\,7610906837\,8941120635\,3368474791\,2954208389\\&7263352003\,7686275679\,0996831645\,2222918013\,3822749913\,\\&1527755618\,1523970004\,1829353798\,5819153203\,\\&8804954205\,2390123411\,59169\pm 10^{-165}. \end{aligned}$$

Using the Monte-Carlo method with $N_{mc}=1000$ pseudo-random points in the interval [0, 1] and $t_{mc}=100$ samples, we can numerically check the output of the routine MaxValue. Namely, taking the maximum of the ratios $\frac{{\mathcal {L}}_{1+\varepsilon } p}{p}$ and $\frac{{\mathcal {L}}_{1-\varepsilon }q}{q}$ computed at 1000 different points a hundred times, we obtain the values which lie within the distance of $2.0\times 10^{-345}$ from $\alpha$ or $\beta$, respectively.

### A family of full branch piecewise Möbius maps

We next consider a family of examples studied by Slipantschuk, Bandtlow and Just in [19] in connection with their study of relation between Lyapunov exponents and mixing rates.

Following [19], for $- \frac{1}{4} \le c \le \frac{1}{2}$ we have a map $f_c :[-1,1] \rightarrow [-1,1]$ defined by

$$\begin{aligned} f_c(x) = \frac{1-2(c+1)|x|}{1 + 2 c |x|}. \end{aligned}$$

**Fig. 3:**
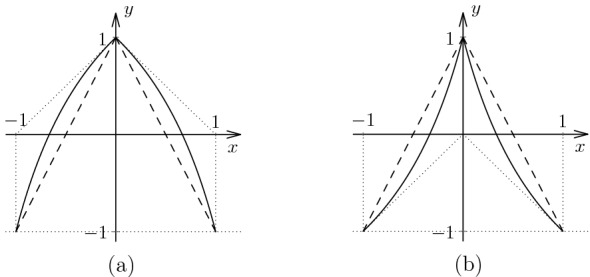
Two plots of the bent tent map for parameter values close to the ends of the parameter interval: $c = 0.2495$ (**a**) and $c = 0.4995$ (**b**). The dashed lines are the tent map corresponding to $c=0$

When $c=0$ this reduces to a piecewise linear “tent map” (see Fig. 3 for a plot). In the special case $c=0.11$, of particular importance to the authors of [19], they assert that the Lyapunov exponent is $\lambda (f_{0.11},\mu _{0.11}) = 0.685 \ldots$, although the paper does not provide any details as to how this value was computed.

The inverse branches $f_1, f_2: [-1,1] \rightarrow [-1,1]$ take the form

$$\begin{aligned} f_1(x) = \frac{1-x}{2cx + 2(c+1)} \qquad \text{ and } \qquad f_2(x) = - \frac{1-x}{2cx + 2(c+1)}. \end{aligned}$$

In particular, $|f_1'(x)| = |f_2'(x)| = \frac{1+2c}{2(1 + c + x )^2}$. The associated transfer operator is given by

$$\begin{aligned} \left[ {\mathcal {L}}_t h \right] (x){} & {} = \left| \frac{2 (2c+1)}{(2c x + 2(c+1)^2)}\right| ^t h\left( \frac{1-x}{2cx + 2(c+1)} \right) \\{} & {} \quad + \left| \frac{2 (2c+1)}{(2c x + 2(c+1)^2)}\right| ^t h\left( - \frac{1-x}{2cx + 2(c+1)} \right) . \end{aligned}$$

We shall recover and improve the estimate of [19]. For this purpose, we choose $m=400$ and compute Chebyshev nodes with accuracy of 600 digits. Then we choose $\varepsilon = 10^{-175}$ and apply Chebyshev collocation method to obtain two polynomials *p* and *q* of degree 399. We then verify that they are positive and evaluate

$$\begin{aligned} \alpha : = -\log \left( {\texttt { MaxValue}} \frac{\mathcal L_{1+\varepsilon } p}{p}\right) \text{ and } \beta : = \log \left( {\texttt { MaxValue}} \frac{{\mathcal {L}}_{1-\varepsilon } q}{q} \right) . \end{aligned}$$

with working precision set to 400. For each of the values we give 180 digits.

$$\begin{aligned} \alpha = 0.&6849333272\, 2256432968\, 5622546648\, 2230532357\, 7867689297\, 3987148578\,\\&8085505250\, 5345328689\, 5040861069\, 9964717724\, 0662692746\, 4804164759\,\\&1723161867\, 2782003116\, 7550103160\, 3289137884\, 1128687391\,\\&8360864512 \times 10^{-175}; \\ \beta = 0.&6849333272\, 2256432968\, 5622546648\, 2230532357\, 7867689297\, 3987148578\,\\&8085505250\, 5345328689\, 5040861069\, 9964717724\, 0662692746\, 4804164759\,\\&1723161867\, 2782003116\, 7550103160\, 3289137884\, 1128687391\,\\&8360866430 \times 10^{-175}. \end{aligned}$$

This yields the following estimate on the Lyapunov exponent accurate to 176 decimal places given below:

$$\begin{aligned} \begin{aligned} \lambda (f_{0.11},\mu _{0.11}) =\,&0.6849333272\, 2256432968\, 5622546648\, 2230532357\,\\&7867689297\, 3987148578\,8085505250\, 5345328689\, 5040861069\,\\&9964717724\, 0662692746\, 4804164759\,1723161867\,\\&2782003116\, 7550103160\, 3289137884\, 1128687391\,8360865 \pm 10^{-177}. \end{aligned} \end{aligned}$$

**Fig. 4:**
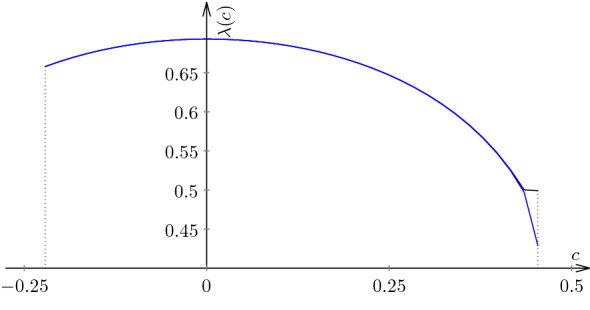
Lower (dashed curve) and upper (solid curve) bounds on the Lyapunov exponent for the family of bent tent maps. We see that for $c \in (-0.24, 0.42)$ they are almost indisitnguishable. This is the range of parameter values where our method is particularly effective

In addition, similarly to the case of the Lanford map, we can plot the Lyapunov exponent as a function of the parameter *c*. A sketch of the graph $\lambda (c)$ is shown in Fig. 4. It is based on the computation for 40 equidistant points in the parameter interval $(-0.24,0.45)$. The following setup has been used for the calculation: $\varepsilon = 0.001$, $N = 128$ Chebyshev nodes computed with accuracy of 512 digits. For the parameter values $c \in (-0.25,-0.24)$ and $c \in (0.45, 0.5)$ the computation turns to be unstable and the resulting values of $\alpha$ and $\beta$ disagree by as much as 1.3 for $c = -0.22$. This is again due to the fact that $|f_c^\prime (0)| \rightarrow 1$ as $c \rightarrow -0.25$ and $|f_c^\prime (\pm 1)|\rightarrow 1$ as $c \rightarrow 0.5$, i.e. diminuishing hyperbolicity of the system.

### Bent baker’s map

**Fig. 5:**
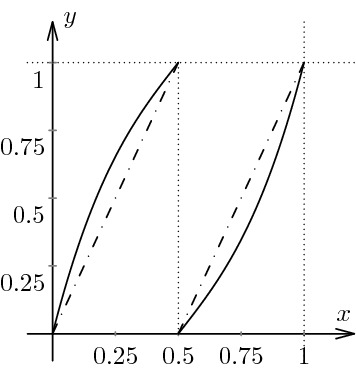
The solid curve is a plot of the bent baker’s map. For comparison, the dashed line is the plot of the doubling map

Finally, we consider an example studied by Froyland in [9] (Fig. 5). Namely, we can consider the map $f:[0,1] \rightarrow [0,1]$ defined by

$$\begin{aligned} f(x) = \frac{4\sqrt{6}}{3}x^3 - 2 \sqrt{6} x^2 + \left( 2 + \frac{2\sqrt{6}}{3}\right) x \quad \text{ mod } 1. \end{aligned}$$

The inverse branches $f_1, f_2: I \rightarrow I$ are defined by

$$\begin{aligned} f_1(x)&= \frac{-2 + 2\sqrt{6}+ 2^{2/3}\left( 9x + \sqrt{-38 + 18 \sqrt{6} + 81x^2}\right) ^{2/3}}{2^{11/6} \sqrt{-\frac{38}{3} + 6^{3/2} + 27 x^2} \left( 9x + \sqrt{-38 + 18\sqrt{6} + 81x^2} \right) ^{1/3}} \\ f_2(x)&= \frac{-2 + 2\sqrt{6}+ 2^{2/3}\left( -9 + 9x + \sqrt{43 + 18\sqrt{6} - 162 x+ 81x^2} \right) ^{2/3}}{2^{11/6} \sqrt{-\frac{43}{3} + 6^{3/2} -54 x + 27 x^2} \left( -9x + \sqrt{43 + 18\sqrt{6} - 162 x + 81x^2} \right) ^{1/3}} \end{aligned}$$

and we can associate the transfer operators ${\mathcal {L}}_t$ for $t \in {\mathbb {R}}$ according to (1). We next want to choose the following parameters for the computation. First, we compute $m=129$ Chebyshev nodes with accuracy of 512 digits. Then we fix $\varepsilon = 10^{-75}$ and compute two polynomials *p* and *q* using Chebyshev collocation method. Afterwards, we use working precision of 256 for the routines MinValue and MaxValue. The calculation gives

$$\begin{aligned} \alpha&= -\log \left( {\texttt { MaxValue}} \frac{\mathcal L_{1+\varepsilon } p}{p} \right) \\&= 0.6494631493\, 2069852907\, 6505\ldots \times 10^{-75}; \quad \text{ and } \\ \beta&= \log \left( {\texttt { MinValue}} \frac{\mathcal L_{1-\varepsilon } q}{q}\right) \\&= 0.6494631493\, 2069852907\, 7088 \ldots \times 10^{-75}. \end{aligned}$$

We obtain the value of the Lyapunov exponent

$$\begin{aligned} \lambda (f,\mu ) = 0.6494631493\,2069852907\, 6 \pm 10^{-21}. \end{aligned}$$

This is consistent with, and improves on, Froyland’s estimate of $\lambda (f,\mu ) = 0.64946$. We see that in this case the accuracy is less than in other examples we have considered so far. One cause is the character of the inverse branches $f_1$ and $f_2$: the formulae implies that providing we know the value of $x\in (0,1)$ with an error of $10^{-k}$, we have the value of $f_{1}(x)$ and $f_{2}(x)$ with an error of $10^{-k/6}$.

Another source of complication is the diminished hyperbolicity. A straightforward calculation gives that $f'(x) \ge f'(\frac{1}{2}) = 2 + 2 \sqrt{\frac{2}{3}} - \sqrt{6} =1.1835\ldots$. This relatively weak hyperbolicity also suggests an explanation for why the estimates are not as good as in the previous examples. In particular, the maximal eigenfunction for ${\mathcal {L}}_t$ may be less regular (e.g., analytic on a relatively small Bernstein ellipse) which make the polynomial approximation used in §4.2 less effective.

## Proof of Theorem 1.4

In order to explain the proof of Theorem 1.4 it helps to introduce the following famous function from thermodynamic formalism.

### Pressure function

We begin by introducing the following well known definition.

#### Definition 3.1

To any map $f \in NC(I)$ we can associate the *pressure function* $P:{\mathbb {R}} \rightarrow {\mathbb {R}}$ defined by

$$\begin{aligned} P(t) = \lim _{n\rightarrow +\infty } \frac{1}{n} \log \sum _{f^nx=x} |(f^n)'(x)|^{-t} \hbox { for } t \in {\mathbb {R}}, \end{aligned}$$

where the summation is taken over all fixed points for the iterates $f^n$, $n \ge 1$.

This is one of many equivalent definitions of the pressure [21]. In fact, the pressure function is well defined in the setting of continuous maps. However, the method we give in the present manuscript requires the pressure function to be differentiable in a neighbourhood of 1. The usefulness of the pressure function to study the Lyapunov exponent is shown by the following simple lemma, the first three parts of which are well-known.

#### Lemma 3.2

The pressure function has the following properties:

1. $$P(1) = 0$$;
2. *P* is an analytic convex function;
3. We can write $\lambda (f, \mu ) = - \frac{dP(t)}{dt}|_{t=1}$, where $\mu$ is absolutely continuous *f*-invariant probability measure, and;
4. For any $\varepsilon > 0$ we can write
  $$\begin{aligned} -\frac{P(1+\varepsilon )}{\varepsilon } \le - \frac{dP(t)}{dt}\Bigl |_{t=1} \le \frac{P(1-\varepsilon )}{\varepsilon }. \end{aligned}$$

#### Proof

**Fig. 6:**
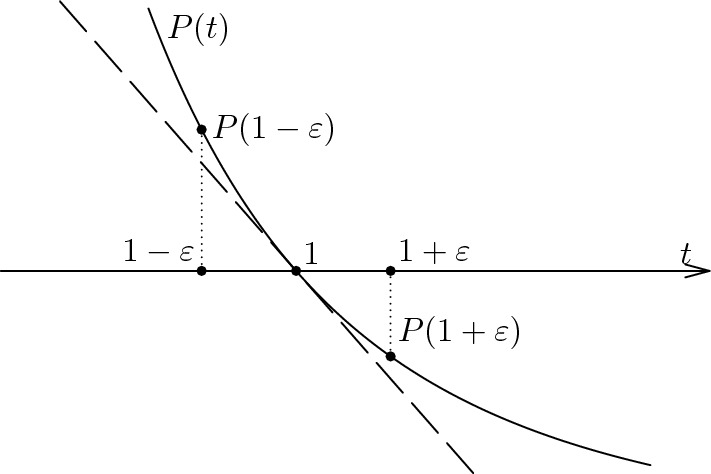
The pressure function *P*(*t*) and the inequalities in part (4) of the Lemma

The first three parts are essentially due to Ruelle [18] (see Corollary 5.27 and Exercise 5 (a) on p.99). The last observation follows easily from the convexity (see Fig. 6). $\square$

This leads to the following useful bound on the Lyapunov exponent.

#### Corollary 3.3

For any $\varepsilon > 0$ the following double inequality holds

$$\begin{aligned} -\frac{P(1+\varepsilon )}{\varepsilon } \le \lambda (f, \mu ) \le \frac{P(1-\varepsilon )}{\varepsilon }. \end{aligned}$$

#### Proof

This comes by substituting the identity in part 3 of Lemma 3.2 into the inequality in part 4. $\square$

#### Remark 3.4

At first sight, it may not seem very promising as an approach to estimating $\lambda (f,\mu )$ to have to compute the pressures $P(1\pm \varepsilon )$ with an error $O(\varepsilon ^2)$ in order to have an estimate on $\lambda (f,\mu )$ with error $O(\varepsilon )$. This means that one has to estimate $P(1\pm \varepsilon )$ with the double accuracy of the desired estimate for the Lyapunov exponent. Nevertheless it turns out that this approach is quite practical since it is quite easy to estimate the pressure to high precision.

### Transfer operator for interval maps

For definiteness, let us choose coordinates such that $I = [-1,1]$ (i.e., $a=-1$ and $b=1$ in Definition 1.1) after a simple change of coordinates. In addition, we shall also assume for simplicity that the map *f* is full-branch, i.e., $f((x_k, x_{k+1})) = (-1,1)$, with inverse branches $f_k: (-1,1) \rightarrow (x_k, x_{k+1})$, for $k=1, \ldots , n$, the general case being similar.

The approach to estimating the pressure is based on its interpretation in terms of the family of transfer operators introduced in the Introduction. These operators act on the Banach space ${\mathcal {B}}$ of bounded analytic functions on domain $U_\rho \supset I$ enclosed by the Bernstein ellipse with the foci at $-1$ and 1 and given by

$$\begin{aligned} \partial U_\rho = \left\{ z = \frac{1}{2} \left( \rho e^{i \theta } + \frac{e^{-i\theta }}{\rho }\right) :0 \le \theta < 2\pi \right\} ,\quad \rho > 1, \end{aligned}$$

We define the norm on ${\mathcal {B}}$ by $\Vert f\Vert = \sup _{z\in U} |f(z)|$. In particular, by choosing $\rho$ sufficiently close to 1 we can assume that the inverse branches $f_j$ ($j=1, \ldots , n$) of the map $f \in NC(I)$ have analytic extensions to $U_\rho$. Therefore the maps $f_j: U_\rho \rightarrow U_\rho$ are well defined and their derivatives are non-zero. Furthermore since all $f_j$ are contractions, we may assume without loss of generality that $\overline{\cup _j f_j U_\rho } \subset U_\rho$ otherwise we can replace *f* by an iterate $f^n$, where *n* is chosen sufficiently large that the condition holds with the inverse branches $f_j$ for *f* replaced by the inverse branches of $f^n$. Of course, this would increase the number of contractions we are working with. We formally extend the definition of the transfer operators from (2) as follows:

#### Definition 3.5

A family of transfer operators ${\mathcal {L}}_t: {\mathcal {B}} \rightarrow {\mathcal {B}}$ associated to $f \in NC(I)$ is defined by

6 $$\begin{aligned} {[}{\mathcal {L}}_t h](x) = \sum _{k=1}^n |f_{j}^\prime (x)|^t h(f_{j}(x)) \qquad \text{ for } x\in U_\rho , \text{ and } t \in {\mathbb {R}}. \end{aligned}$$

#### Remark 3.6

In this definition the functions $$|f_j'|^{t}$$ are real valued and real analytic on $$[-1,1]$$. Thus by a slight abuse of notation we interpret $$|f_j'|^{t} $$ as being the complex analytic extension of these functions to $$U_\rho $$.

We can estimate the pressure values *P*(*t*) using the maximal eigenvalue for the transfer operator ${\mathcal {L}}_t$.

#### Lemma 3.7

Let $f \in NC(I)$ and let ${\mathcal {L}}_t :{\mathcal {B}} \rightarrow {\mathcal {B}}$ be the transfer operator defined by (6). Then

1. The spectral radius of ${\mathcal {L}}_t$ is $e^{P(t)}$.
2. The rest of the spectrum is contained in a disk of radius strictly smaller than $e^{P(t)}$.
3. For any $h \in {\mathcal {B}}$ for which the restriction to *I* is strictly positive and any $x \in I$ we have $\lim _{n \rightarrow +\infty } \left( {\mathcal {L}}_t^n h(x) \right) ^{\frac{1}{n}} = e^{P(t)}$.

#### Proof

Parts 1 and 2 are essentially due to Ruelle [18] (see Proposition 5.13 and 5.24). Part 3 follows directly from Part 1 and Part 2 and the classical spectral radius theorem (cf. [18], Proposition 5.13 and 5.14). Another exposition can be found in [16]. $\square$

We can use this lemma to estimate the pressure values $P(1\pm \varepsilon )$ in Corollary 3.3. In particular, in order to estimate $e^{P(t)}$ for $t \in {\mathbb {R}}$ we will use the following simple result.

#### Lemma 3.8

Assume that for $t \in {\mathbb {R}}$ there exist a function $p \in {\mathcal {B}}$, strictly positive on *I*, and a constant $\rho \in {\mathbb {R}}$ such that $\sup _{x \in I}\frac{ {\mathcal {L}}_{t} p(x)}{p(x) } \le e^{\rho }$ then $e^{P(t)} \le e^{\rho }$.

#### Proof

Since ${\mathcal {L}}_{t} p(x) \le e^{\rho } p(x)$ for all $x \in I$ we can deduce that ${\mathcal {L}}_{t}^n p(x) \le e^{n \rho } p(x)$ for all $n \ge 1$. Thus by Part 3 of Lemma 3.7 we have $e^{P(t)} = \lim _{n\rightarrow +\infty } \left( {\mathcal {L}}_{t}^n p (x) \right) ^\frac{1}{n} \le e^\rho$ for any $x\in I$. $\square$

We now combine Lemma 3.8 and Corollary 3.3 to prove Theorem 1.4.

#### Proof of Theorem 1.4

By assumption, we know that there exist $0< \alpha < \beta$ and two positive functions *p* and *q* such that

$$\begin{aligned} \sup _I \frac{{\mathcal {L}}_{1+\varepsilon } p}{p} \le e^{-\alpha } \hbox { and } \sup _I \frac{{\mathcal {L}}_{1-\varepsilon } q}{q} \le e^{\beta }. \end{aligned}$$

Applying Lemma 3.8 with $t=1+\varepsilon$ and $\rho =-\alpha$ then gives $e^{P(1+\varepsilon )} \le e^{-\alpha }$ and thus $P(1+\varepsilon ) \le -\alpha$, or equivalently, $-P(1+\varepsilon ) \ge \alpha$. On the other hand, applying Lemma 3.8 with $t=1-\varepsilon$ and $\rho =\beta$ then gives $e^{P(1-\varepsilon )} \le e^\beta$ and thus $P(1-\varepsilon ) \le \beta$. Combining these two inequalities with Corollary 3.3 we get $- \frac{ \alpha }{\varepsilon }\le \lambda (f, \mu ) \le \frac{ \beta }{\varepsilon }$, as required. $\square$

#### Remark 3.9

The above arguments extend easily to all maps $f \in NC(I)$, not necessary full branch. In particular, instead of a single domain $U_\rho$, we consider the disjoint union $U = \coprod _{k=1}^n U_\rho ^{(k)}$ of domains $U_\rho ^{(k)} \supset [x_k, x_{k+1}]$ each bounded by a Bernstein ellipse with foci $x_n$ and $x_{n+1}$ ($k=1, \cdots , n$). The Banach space is now taken to be ${\mathcal {B}} = \oplus _{k=1}^n {\mathcal {B}}^{(k)}$ where $\mathcal B^{(k)}$ is the space of bounded analytic functions on $U_\rho ^{(k)}$. Finally, we use the extension of (1) to define ${\mathcal {L}}_t: {\mathcal {B}} \rightarrow {\mathcal {B}}$ by

$$\begin{aligned} {[}{\mathcal {L}}_t {\underline{h}}]_j(x) = \sum _{k: f((x_k,x_{k+1})) \cap (x_j,x_{j+1}) \ne \varnothing } |f_{jk}^\prime (x)|^t h_k(f_{jk}(x)) \qquad t \in {\mathbb {R}}, x\in U_\rho ^{(j)}. \end{aligned}$$

where ${\underline{h}} = (h_1, \ldots , h_n) \in {\mathcal {B}}$. The argument then proceeds as above.

## Practical realisation

We next want to explain how to apply Theorem 1.4 in practice. Below we give one way of constructing test functions *p* and *q* that we used in order to obtain estimates in the examples we considered. It is based on the spectral Chebyshev collocation method [20]. There are other methods one might consider, such as spline interpolation methods, proposed by Falk and Nussbaum [8], but this approach suffices for our needs.

A particular care is needed in implementation of the algorithm to avoid numerical errors. We implemented the method using the Mathematica built-in functions which use interval arithmetic to control possible errors. The method itself however can be implemented in any programming language where there libraries for interval arithmetic are readily available, e.g. C, Julia, or Python (in alphabetic order).

### Constructing test functions *p* and *q*

For notational simplicity, we will describe our construction in the special case of full branch maps. The generalization to the general case of Markov maps is fairly straightforward where *I* is replaced by the disjoint union of intervals.

#### Definition 4.1

Let $x_1< x_2< \cdots < x_n$ be a collection of *n* distinct real numbers. The Lagrange polynomials associated to $\{x_j\}_{j=1}^n$ are the polynomials

7 $$\begin{aligned} \ell p_j(x): = \prod _{k\ne j} \frac{x-x_k}{x_j-x_k}, \qquad j = 1, \ldots , m. \end{aligned}$$

The Lagrange polynomials have the property that $\ell p_j(x_k) = \delta _j^k$ for all $j = 1, \ldots , m$ and $k = 1, \ldots , n$. In a special case when the points $\{x_j\}_{j=1}^n$ are the roots of a certain polynomial $p_n$, they can be written as

8 $$\begin{aligned} \ell p_j(x) = p_n(x) \cdot (p_n^\prime (x_j))^{-1} \cdot (x-x_j)^{-1}, \qquad j = 1, \ldots , m. \end{aligned}$$

We assume below that $I = [-1,1]$, the general case being similar after a simple change of coordinates, and $f \in NC(I)$. Let us assume that one wishes to compute the Lyapunov exponent with an error of $\delta$, in other words, we assume that one wishes to find an interval $(\lambda _1,\lambda _2) \ni \lambda (f,\mu )$ such that $|\lambda _2 - \lambda _1| \le \delta$. In order to define the functions *p* and *q*, we begin by choosing a natural number $m = m(\delta )$. Then we calculate numerically, with help of a computer, the following objects:

1. Chebyshev nodes $x_k: = \cos \left( \frac{\pi (2k+1)}{2m}\right) \in (-1,1)$, for $k = 0,\ldots ,m-1$ — these are the roots of the Chebyshev polynomial of the first kind $T_m$. In a general case of $I=[a,b]$ the Chebyshev nodes have to be rescaled and shifted to *I* using the transformation $x\mapsto x(b-a)+a$.
  The cosine function can be evaluated at a given point with arbitrary precision. In particular, in each of the Examples we consider we specify the number of digits $N = N(\delta )$ requested in the actual program code.
2. For $t = 1 \pm \varepsilon$ the matrices $M^t \in GL(m,{\mathbb {R}})$ given by
  9 $$\begin{aligned} M^t_{jk} :=[{\mathcal {L}}_t \ell p_j](x_k) = \sum _{i = 1}^m |f_i^\prime (x_k) |^{t} \cdot (\ell p_j( f_i(x_k)); \end{aligned}$$
  Here we use the formula (8) to evaluate $\ell p_j( f_i(x_k))$, using an inbuilt routine for evaluation of Chebyshev polynomials, which has guaranteed accuracy.
3. The leading left eigenvectors $v^t$ corresponding to the maximal eigenvalue of the matrices $M^t$ for $t = 1 \pm \varepsilon$. They can be efficiently computed using the power method.
4. The polynomials *p* and *q* then given by linear combinations of Lagrange polynomials $\ell p_j$ with coefficients coming from the eigenvectors:
  10 $$\begin{aligned} p(x) = \sum _{j=0}^{m-1} v_j^{(1-\varepsilon )} \ell p_j(x) \qquad \text{ and } \qquad q(x) = \sum _{j=0}^{m-1} v_j^{(1+\varepsilon )} \ell p_j(x). \end{aligned}$$
  However, the formula (10) is prone to numerical errors. The polynomials *p* and *q* can also be written as a linear combination of Chebyshev polynomials, and this has the advantage of being more computationally stable than the more direct expansion in terms of Lagrange polynomials above. More precisely, the following expansion is well known.
  $$\begin{aligned} p(x) = \sum _{j=0}^{m-1} a_j T_j(x), \qquad \text{ and } q(x) = \sum _{j=0}^{m-1} b_j T_j(x), \end{aligned}$$
  where the coefficients are given in terms of the eigenvectors $v^{(1-\varepsilon )}$ and $v^{(1+\varepsilon )}$:
  $$\begin{aligned} a_j&= \frac{2}{m} \sum _{k=1}^m v_k^{(1-\varepsilon )} T_j(x_k){} & {} \text{ and } \quad b_j = \frac{2}{m} \sum _{k=1}^m v_k^{(1+\varepsilon )} T_j(x_k), \text{ for } j = 1, \dots , m-1; \\ a_0&= \frac{1}{m} \sum _{k=1}^m v_k^{(1-\varepsilon )}{} & {} \text{ and } \quad b_0 = \frac{1}{m} \sum _{k=1}^m v_k^{(1+\varepsilon )}. \end{aligned}$$
  This allows us to evaluate *p* and *q* efficiently.
5. The supremums of the ratios $\frac{{\mathcal {L}}_{1+\varepsilon } p}{p}$ and $\frac{{\mathcal {L}}_{1-\varepsilon } q}{q}$ over the interval *I* is computed using internal routine MaxValue with working precision set to $D=D(\delta )$ digits.

In addition to exploiting the internal routine MaxValue we can also apply Monte Carlo type method in order to carry out a heuristic check on its output.

In the next subsection we show that in the setting of uniformly expanding piecewise analytic Markov maps the polynomials *p* and *q* satisfying the hypothesis of Theorem 1.4 can always be constructed, and moreover the conclusion of Theorem 1.7 holds.

### Justification of the method: Proof of Theorem 1.7

We can denote by ${\mathcal {P}}_m \subset {\mathcal {B}}$ the space of polynomials on $I = [-1,1]$ of degree not greater than *m*. We let $\pi _m: {\mathcal {B}} \rightarrow {\mathcal {P}}_m$ be the projection onto the polynomials of degree *m* given by the Chebychev–Lagrange collocation formula

$$\begin{aligned} \pi _m(f)(x) = \sum _{j=0}^m f(x_j) \ell p_j(x), x \in I, \end{aligned}$$

where $x_j$, $j = 0, \ldots , m$ are the roots of the Chebyshev polynomial $T_{m+1}$, i.e. the Chebyshev nodes and $\ell p_j$ ($j=0, \cdots , m$) are the Lagrange polynomials on *I* associated to $x_j$, $j = 0, \ldots , m$ defined by (7). In particular, we see that the restriction $\pi _m|_{{\mathcal {P}}_m}$ of $\pi _m$ to ${\mathcal {P}}_m$ is the identity.

The transfer operator ${\mathcal {L}}_{t}:{\mathcal {B}} \rightarrow {\mathcal {B}}$ defined by (6) is compact, even nuclear, although this will not be needed. We require an estimate on the operator norm of the difference ${\mathcal {L}}_t - {\mathcal {L}}_t \pi _m$ defined by

$$\begin{aligned} \Vert {\mathcal {L}}_t - {\mathcal {L}}_t \pi _m\Vert = \sup _{\Vert f\Vert _{{\mathcal {B}}}=1} \Vert ({\mathcal {L}}_t - {\mathcal {L}}_t \pi _m) (f) \Vert _{{\mathcal {B}}}. \end{aligned}$$

#### Lemma 4.2

(see [4], Theorem 3.3). Let $f \in NC(I)$ and let ${\mathcal {L}}_t$ be the associated transfer operator defined by (6). Then there exists $C > 0$ and $0< \theta < 1$ such we can bound that $\Vert {\mathcal {L}}_t - \mathcal L_t \pi _m\Vert \le C \Vert {\mathcal {L}}_t\Vert \theta ^m$ for $m \ge 1$.

This can be compared to [22, §2.2] where it is shown that $\Vert {\mathcal {L}}_t \pi _m - \pi _m {\mathcal {L}}_t \pi _m \Vert _{BV} \rightarrow 0$ as $m\rightarrow \infty$ using the bounded variation norm.

#### Remark 4.3

Although we cannot expect that $\Vert I - \pi _m\Vert _{{\mathcal {B}} \rightarrow {\mathcal {B}}} \rightarrow 0$ as $m\rightarrow \infty$, the composition with the operator ${\mathcal {L}}_t$ allows the bound in Lemma 4.2 since for any function *f* analytic on $U_\rho$ for some $\rho >1$, there exists $\rho ^\prime > \rho$ such that the image ${\mathcal {L}}_tf$ is analytic on $U_{\rho ^\prime }$.

It follows from the properties of ${\mathcal {L}}_t$, Lemma 4.2 and classical analytic perturbation (see the book of Kato [12]) that we have the following:

#### Lemma 4.4

Let $f \in NC(I)$ and let ${\mathcal {L}}_t$ be the associated transfer operator defined by (6). Then for $\delta >0$ sufficiently small and *m* sufficiently large:

1. ${\mathcal {L}}_t \pi _m: {\mathcal {B}} \rightarrow {\mathcal {B}}$ has a simple maximal eigenvalue $\lambda _m$ with $|\lambda _m - e^{P(t)}| < \delta$;
2. The rest of the spectrum is contained in $\{z\in {\mathbb {C}} :|z|\ < e^{P(t)} - 2\delta \}$; and
3. The corresponding eigenfunction $h_m$ for ${\mathcal {L}}_t \pi _m$ has a restriction to *I* which is strictly positive (i.e, $h_m(x) > 0$ for $x\in I$).

By perturbation theory the positivity of the restriction of the eigenfunction *h* associated to $e^{P(t)}$ for ${\mathcal {L}}_t$ onto *I* implies the same for $h_m$ (since $\inf _{x\in I} |h(x) - h_m(x)| \le \Vert h - h_m\Vert$ will be arbitrary small for *m* sufficiently large). The restriction $\pi _m {\mathcal {L}}_t|_{\mathcal P_m}$ is a finite rank operator $\pi _m {\mathcal {L}}_t: {\mathcal {P}}_m \rightarrow {\mathcal {P}}_m$ given by

$$\begin{aligned} \pi _m {\mathcal {L}}_t : g \mapsto \sum _{j=0}^m [{\mathcal {L}}_t g](x_j) \ell p_{j}, \end{aligned}$$

where $x_j$, $j = 0, \ldots , m$ are the Chebyshev nodes introduced in Sect. 4.1. Observe that in the basis of Lagrange polynomials $\{\ell p_j\}_{j=0}^m$ given by (7) the operator $\pi _m {\mathcal {L}}_t$ is given by the matrix $M^t$ defined by (9).

In particular, maximal eigenvalue $\lambda _m$ for $\mathcal L_t\pi _m$ is also an eigenvalue for the matrix $M^t$ corresponding to the eigenvector $\pi _m(h_m) \in {\mathcal {P}}_m$. This completes the proof of Theorem 1.7. $\square$

Given $f \in NC(I)$ and $\delta >0$ there exist $N = N(\delta )$ and $D = D(\delta )$ such that the polynomials *p* and *q* of degree $m \ge N$ with coefficients computed to *D* decimal places lead to estimates with $\frac{1}{\varepsilon }\log \frac{\beta }{\alpha }< \delta$. In particular, the exponential convergence in Lemma 4.2 implies that $N(\delta ) = O(\log (\delta /\varepsilon ))$ and $D(\delta ) = O(-\log \delta , -\log \varepsilon )$.

#### Remark 4.5

(Heuristic estimates on the accuracy of approximation) For small $|\varepsilon |\ll 1$ an $O(\varepsilon ^2)$ approximation to the eigenvalue $e^{P(t)}$ should give an $O(\varepsilon )$ estimate on the Lyapunov exponent (since we divide out by $\varepsilon$ in the formulae). Furthermore, this error is related to the (uniform) approximation error of the associated eigenfunction *f* by the interpolating polynomial based on *m* points, say, which is well known to be bounded by $\Vert f^{k}\Vert _\infty (k+1)!$. Even for very regular (e.g., analytic) functions *f* one only expects $\Vert f^{k}\Vert _\infty$ to tend to zero at best exponentially fast. Therefore, we might want $\varepsilon \sim \sqrt{1/(k+1)!}$. In particular, for degree $k=10$ one gets $\varepsilon = 5.10^{-4}$, for $k=20$ one gets $\varepsilon =6.10^{-10}$, and

for $k=100$ one gets $\varepsilon =1.10^{-79}$.

### Potential generalisations

In principle the method in this paper can be used in more general settings. It should apply with a few modifications to Markov expanding maps in arbitrary dimensions.

However, relaxing the Markov condition may present serious difficulties. Whereas the properties of the pressure in §3.1 persist in the case when $\log |f^\prime |$ is a function of bounded variation the construction of the test functions *p* and *q* in §4.1 in this space may be more problematic.

Similarly, relaxing the hyperbolicity assumption may prove challenging in this case because of potential lack of differentiability of *P*(*t*) at $t=0$. This happens for instance in the case of phase transition associated to the Manneville–Pomeau and Liverani–Saussol–Vaienti maps [15].

## Footnotes

1 This is equivalent to the map being locally eventually onto, i.e. to saying that there exists $N \ge 1$ such that for each $1\le j \le n$ we have $\overline{f^N( (x_j,x_{j+1}) )} = I$.

2 The reason for this choice of notation is that in practice the functions *p* and *q* are polynomials.
